# Association of healthy lifestyle with tooth count among adults aged 20 years and above: a cross-sectional study

**DOI:** 10.1186/s12903-026-08298-3

**Published:** 2026-04-13

**Authors:** Zheng Zhang, Chen Qu, Chunxian Lv, Zhiyuan Jin, Tao Mao, Furu Wang, Zhi Zhang, Zhida Sun

**Affiliations:** 1 Department of Oral Health Management Center, Affiliated Stomatological Hospital of Nanjing Medical University, Nanjing, China; 2 Department of Health Education, Jiangsu Provincial Center for Disease Control and Prevention, Nanjing, China; 3 Department of Psychiatry, Third People’s Hospital of Zhongshan City, Zhongshan, China; 4 Medical Prevention Coordination Management Office, Jiangsu Provincial Center for Disease Control and Prevention, Nanjing, China; 5 Department of Health Management Service, Jiangsu Provincial Center for Disease Prevention and Control, Nanjing, China

**Keywords:** Healthy lifestyle, Teeth, NHANES, Smoking

## Abstract

**Background:**

While healthy lifestyle has been extensively studied in relation to chronic non-communicable diseases and lifespan, its impact on oral health remains insufficient. This study aimed to examine the association between healthy lifestyle and tooth count, and identify key risk factors.

**Methods:**

Data from 18,919 adults aged ≥ 20 years in the National Health and Nutrition Examination Survey (NHANES) 2005–2018 were analyzed. Participants were categorized into low, medium, and high adherence groups based on their compliance with healthy lifestyle factors. Multiple linear regression and restricted cubic splines were used to assess associations between healthy lifestyle scores and tooth count. Subgroup analyses were performed to identify sensitive populations. Subsequently, five indices, each excluding one different lifestyle factor, were constructed to calculate the strength of their associations with tooth count and to assess the key influencing factors.

**Results:**

After adjusting for covariates, the medium (*β* = 1.13, 95%*CI* = 0.77, 1.49) and high adherence groups (*β* = 1.65, 95%*CI* = 1.20, 2.09) had significantly more teeth than the low adherence group. Each 1-unit increase in the healthy lifestyle score was associated with a 0.49 (95%*CI*: 0.37, 0.61) increase in tooth count. Results from restricted cubic splines confirmed this trend. The association was strongest in adults aged ≥ 65 years (*P*_for interaction_ <0.01). Removing smoking from the lifestyle score attenuated the association most prominently.

**Conclusions:**

Healthy lifestyle is significantly associated with retaining more teeth among the American population, particularly among older adults. Smoking cessation appears to be a critical modifiable factor for preserving tooth count.

**Supplementary Information:**

The online version contains supplementary material available at 10.1186/s12903-026-08298-3.

## Introduction

The number of functional teeth serves as a key indicator of oral function and a critical determinant of quality of life in the population. A reduced number of natural teeth may impair masticatory and grinding functions, which can lead to unfavorable changes in dietary choices. For example, affected individuals tend to avoid hard foods such as certain types of meat and fruits, resulting in decreased intake of essential nutrients and thus adversely affecting nutritional status. Particularly among older adults, those with poor oral function are at a higher risk of developing frailty [1]. Tooth loss can lead to alveolar bone resorption and adjacent tooth tilting, compromising facial aesthetics. Multiple studies have documented significant associations between tooth count and cardiovascular disease, accelerated aging, and all-cause mortality [2–4]. The 2021 Global Burden of Disease (GBD) Study estimated that the age-standardized prevalence rate of major oral diseases was 45,900 cases per 100,000 population, with a total of 3.69 billion people affected globally [5]. Data from China’s Fourth National Oral Health Survey revealed that 84.4% of Chinese adults had dentition defects, with adults aged 65–74 years experiencing an average of 9.5 missing teeth [6]. This age group also showed higher rates of gingival bleeding, dental calculus detection, and attachment loss of ≥ 4 mm compared to other age cohorts. Given the close interconnection between oral health and systemic health, identifying factors influencing tooth count is crucial for improving both oral and overall health outcomes.

Most existing studies have focused on the beneficial effects of healthy lifestyles on outcomes such as chronic diseases, aging and mortality [7, 8], with limited attention to oral health outcomes. Previous research has found that a combination of four lifestyle factors is significantly associated with lower periodontitis prevalence [9]. Dental caries is an important risk factor for tooth loss. A study by Jiang et al. demonstrated significant associations between dental caries and the occurrence of tooth loss in three cross-sectional analyses conducted in 1995, 2005, and 2015 [10]. Furthermore, a healthy eating index (HEI) was found to have a protective effect against dental caries [11]. With advancing age, teeth and periodontal tissues undergo age-related changes including gingival recession and tooth enamel wear, which increase the risk of tooth loss. However, tooth loss is also influenced by unhealthy habits and environmental factors such as heavy metals and polycyclic aromatic hydrocarbons can significantly elevate periodontitis risk [12, 13]. Exploring modifiable lifestyle factors holds great significance for enhancing oral health function and can provide evidence basis for the primary prevention of oral diseases.

To address this research gap, this study examined the association between healthy lifestyle factors and tooth count using data from the National Health and Nutrition Examination Survey (NHANES) database spanning 2005 to 2018.

## Methods

### Study design and population

**Fig. 1:**
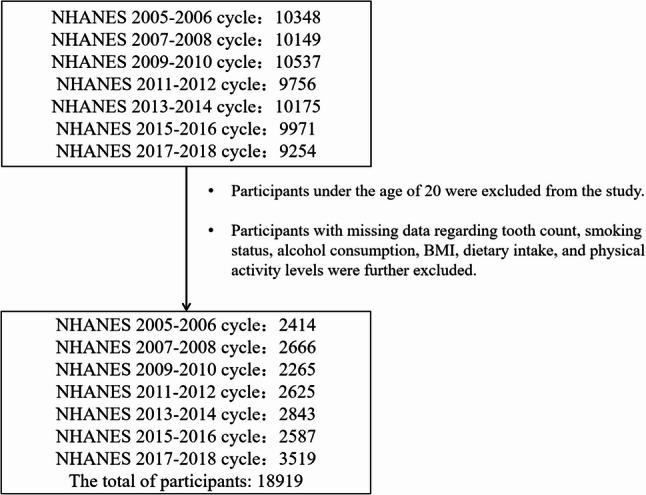
Flow chart of participant selection. NHANES, National Health and Nutrition Examination Survey; BMI, body mass index

This study utilized data from the NHANES conducted between 2005 and 2018. The NHANES database contains information on participants’ basic characteristics, lifestyle factors, and tooth count. As a nationally representative cross-sectional survey designed by the National Center for Health Statistics (NCHS), NHANES employs a complex multistage sampling design. The NCHS Ethics Review Board has reviewed and approved the survey protocol of the NHANES, and all participants have provided their informed consent forms. The study population consisted of adults aged 20 years and above. After excluding individuals under the age of 20 and those with missing data on the five lifestyle factors or the number of teeth, a total of 18,919 participants were included in the final analysis. The number of participants included in the analysis for each cycle of the NHANES from 2005 to 2018 is detailed in Fig. 1.

### Healthy lifestyle assessment

Healthy lifestyle was theoretically defined as a set of health‑promoting behavioral patterns. Based on previous studies, it was operationalized as a composite score integrating five modifiable components: smoking, alcohol consumption, body mass index (BMI), diet, and physical activity [14–17]. Each component was scored according to health criteria, and the total score was used to reflect overall healthy lifestyle level. Information on smoking, alcohol consumption, and physical activity was collected via questionnaires. Dietary data were obtained through two 24-hour dietary recalls; and BMI was calculated using physical examination measurements. A healthy smoking status was defined as having smoked fewer than 100 cigarettes in one’s lifetime. Healthy alcohol intake was defined as ≤ 2 drinks per day for men and ≤ 1 drink per day for women [18]. BMI was calculated as weight (kg) divided by the square of height (m²). A healthy BMI range was 18.5–24.9 kg/m² [19]. Diet quality was assessed using the HEI-2015 score, calculated using the “dietaryindex” package in R software. This index evaluates the adherence of 13 dietary components to recommended dietary guidelines, with a total score ranging from 0 to 100 (higher scores indicate better adherence). A healthy diet was defined as an HEI-2015 score in the highest two quintiles of the study population [20]. Physical activity was quantified by calculating weekly metabolic equivalents of task (MET-min/week), including vigorous and moderate work-related activity, walking or bicycling for transportation, and vigorous and moderate leisure-time physical activity. Physical activity levels were divided into tertiles; the top tertile was considered a healthy level. The healthy lifestyle score ranged from 0 to 5 points, with 1 point assigned for each healthy lifestyle factor and 0 points for unhealthy status (higher scores indicated a healthier lifestyle). Participants were categorized into three groups based on this score: low adherence (0–1 points), moderate adherence (2–3 points), and high adherence (4–5 points).

### Tooth count measurement

Tooth count was defined as the total number of permanent teeth present in the oral cavity. In the NHANES database, dental examination findings are recorded as: primary tooth present, permanent tooth present, dental implant, tooth not present, permanent dental root fragment present. For this study, only the number of present permanent teeth was included in the total tooth count; primary tooth, dental implants and residual roots were excluded.

### Covariates

Covariates included sociodemographic characteristics and chronic disease status. Sociodemographic variables: Gender, age, ethnicity, education level, marital status, and annual household income. Ethnicity was categorized according to NHANES questionnaires: Mexican Americans, other Hispanics, non-Hispanic Whites, non-Hispanic Blacks, and other races. Education level was divided into three categories: Less than high school, high school graduate or equivalent, and more than high school. Annual household income was grouped as: 0–$19,999, $20,000–$44,999, $45,000–$74,999, and $75,000 and above. Marital status was classified into two categories: Married, and widowed/divorced/separated/never married. The frequency of dental visits was classified based on whether the subjects had visited a dentist in the past year. Chronic disease status was determined based on self-reported physician diagnosis of hypertension, diabetes, cardiovascular disease (CVD), chronic kidney disease (CKD), arthritis, respiratory diseases, and cancer.

### Statistical analyses

Baseline characteristics of participants were presented as mean (standard deviation, SD) for continuous variables and frequency (percentage) for categorical variables. Due to the multi-stage stratified sampling design of NHANES, this study conducted weighting during the data analysis, using the SDMVPSU variable as the main sampling unit, the SDMVSTRA variable as the stratification variable, and the WTMEC2YR variable as the weighting variable. We performed multiple linear regression models to analyze the associations between healthy lifestyle score and tooth count. The results were presented with beta coefficients (*β*) and 95% confidence intervals (95%*CI*). Restricted cubic spline (RCS) analysis was performed to evaluate the dose-response relationship between the healthy lifestyle score and tooth count. The knots in RCS were set at the 10th, 50th, and 90th percentiles, with the 50th percentile designated as the reference value. No covariates were adjusted in Model 1. Model 2 was adjusted for a comprehensive set of covariates, including sociodemographic variables (age, gender, ethnicity, education level, marital status, annual household income) and chronic disease status (hypertension, diabetes).

To assess the relative contribution of individual lifestyle factors to tooth count, five alternative indices were constructed (each excluding one distinct lifestyle factor). Participants were reclassified into three groups (0–1 points, 2 points, 3–4 points) for each alternative index, and analyses were additionally adjusted for the excluded lifestyle factor. Stratified analyses by age and gender were also conducted.

All analyses were performed using SAS (version 9.4; SAS Institute, Inc., Cary, NC, USA) and R (version 4.0; R Development Core Team, Vienna, Austria). A two-tailed *P* < 0.05 was considered statistically significant.

## Results

### Characteristics of participants​

The participants in this study had a mean age of 46.99 (16.98) years, with males accounting for 50.59% of the total. Among all ethnicity groups, non-Hispanic Whites had the highest proportion (71.87%). Based on the healthy lifestyle adherence score, participants were stratified into three groups: the low-adherence group (*n* = 3706), the moderate-adherence group (*n* = 12542), and the high-adherence group (*n* = 2671) (Table 1). Weighted frequencies of the baseline characteristics of the study participants were presented in Table S1.

**Table 1:** The characteristics of participants by healthy lifestyle scores

Characteristics	Total	Healthy lifestyle scores			*P*
		0–1(3706)	2–3(12,542)	4–5(2671)	
Number of teeth, mean (SD)	24.89(8.14)	22.69(9.55)	25.08(7.90)	26.78(5.91)	< 0.01
Age, year, mean (SD)	46.99(16.98)	48.93(16.45)	47.04(16.93)	44.32(17.42)	< 0.01
Gender, No. (%)					< 0.01
Male	9881(50.59)	2074(10.19)	6554(33.67)	1253(6.71)	
Female	9038(49.41)	1632(8.81)	5988(32.15)	1418(8.45)	
Ethnicity, No. (%)					< 0.01
Mexican American	2648(7.28)	456(1.20)	1868(5.08)	324(1.00)	
Other Hispanic	1667(4.90)	255(0.64)	1173(3.46)	239(0.79)	
Non-Hispanic White	8828(71.87)	1943(14.32)	5691(46.70)	1194(10.85)	
Non-Hispanic Black	3934(9.81)	824(1.92)	2660(6.72)	450(1.18)	
Other Race	1842(6.14)	228(0.93)	1150(3.86)	464(1.35)	
Education level, No. (%)					< 0.01
Less than high school	3441(11.37)	921(3.25)	2230(7.22)	290(0.91)	
High school graduate or equivalent	4265(22.06)	1008(5.37)	2823(14.59)	434(2.10)	
More than high school	11,213(66.56)	1777(10.39)	7489(44.01)	1947(12.16)	
Annual household income,$, No. (%)					< 0.01
0-$19,999	3028(10.30)	844(2.82)	1903(6.46)	281(1.02)	
$20,000-$44,999	5772(24.67)	1270(5.49)	3849(16.24)	653(2.95)	
$45,000-$74,999	4503(24.85)	822(4.86)	3063(16.57)	618(3.42)	
$75,000 and above	5616(40.18)	770(5.84)	3727(26.55)	1119(7.78)	
Marital status, No. (%)					< 0.01
Married	9983(57.11)	1776(9.97)	6707(38.14)	1500(9.00)	
Widowed/Divorced/Separated/Never married	8936(42.89)	1930(9.04)	5835(27.68)	1171(6.17)	
Dental visit within the past year, No. (%)					< 0.01
Yes	6514(39.57)	1003(6.14)	4375(26.36)	1136(7.08)	
No	12,405(60.43)	2703(12.87)	8167(39.47)	1535(8.10)	
Hypertension, No. (%)					< 0.01
Yes	6383(30.34)	1583(7.51)	4228(20.21)	572(2.62)	
No	12,536(69.66)	2123(11.50)	8314(45.61)	2099(12.55)	
Diabetes, No. (%)					< 0.01
Yes	2011(8.11)	528(2.18)	1340(5.38)	143(0.56)	
No	16,908(91.89)	3178(16.83)	11,202(60.44)	2528(14.61)	
CKD, No. (%)					< 0.01
Yes	1458(6.18)	377(1.75)	945(3.81)	136(0.62)	
No	17,461(93.82)	3329(17.26)	11,597(62.01)	2535(14.55)	
CVD, No. (%)					< 0.01
Yes	1688(7.08)	518(2.12)	1046(4.41)	124(0.56)	
No	17,231(92.92)	3188(16.89)	11,496(61.42)	2547(14.61)	
Arthritis, No. (%)					< 0.01
Yes	4915(25.11)	1265(6.21)	3195(16.22)	455(2.68)	
No	14,004(74.89)	2441(12.79)	9347(49.60)	2216(12.49)	
Respiratory diseases, No. (%)					< 0.01
Yes	1419(7.14)	494(2.49)	853(4.23)	72(0.42)	
No	17,500(92.86)	3212(16.52)	11,689(61.59)	2599(14.75)	
Cancer, No. (%)					< 0.01
Yes	1803(10.30)	415(2.43)	1165(6.50)	223(1.37)	
No	17,116(89.70)	3291(16.57)	11,377(59.32)	2448(13.81)	

*Abbreviation: SD* Standard deviation, *CKD* Chronic Kidney Disease, *CVD* Cardiovascular diseases

### Weighted associations of healthy lifestyle scores with tooth count

With the low-adherence group as the reference, both the moderate and high adherence groups demonstrated positive associations with tooth count in both Model 1 and Model 2. After adjusting for confounders, the effect sizes in Model 2 were slightly attenuated compared to those in Model 1. In Model 2, the *β* coefficients (95%*CI*) were 1.13(0.77, 1.49) for the moderate-adherence group and 1.65 (1.20, 2.09) for the high-adherence group. Additionally, for each 1-point increase in the healthy lifestyle score, the number of teeth increased by 0.49 (95%*CI*: 0.37, 0.61) (Table 2).​

**Table 2:** Weighted regression coefficients *β*(95%*CI)* of tooth count associated with healthy lifestyle score

	Model 1		Model 2	
	β(95%CI)	*P*	β(95%CI)	*P*
Low adherence group	0.00		0.00	
Moderate adherence group	2.40(1.96, 2.84)	< 0.01	1.13(0.77, 1.49)	< 0.01
High adherence group	4.09(3.59, 4.60)	< 0.01	1.65(1.20, 2.09)	< 0.01
Each additional healthy lifestyle factor	1.22(1.08, 1.36)	< 0.01	0.49(0.37, 0.61)	< 0.01

No covariates were adjusted in Model 1. Model 2 was adjusted for age, gender, ethnicity, education level, marital status, annual household income, dental visit within the past year, hypertension, diabetes, CKD, CVD, arthritis, respiratory diseases, and cancer

**Fig. 2:**
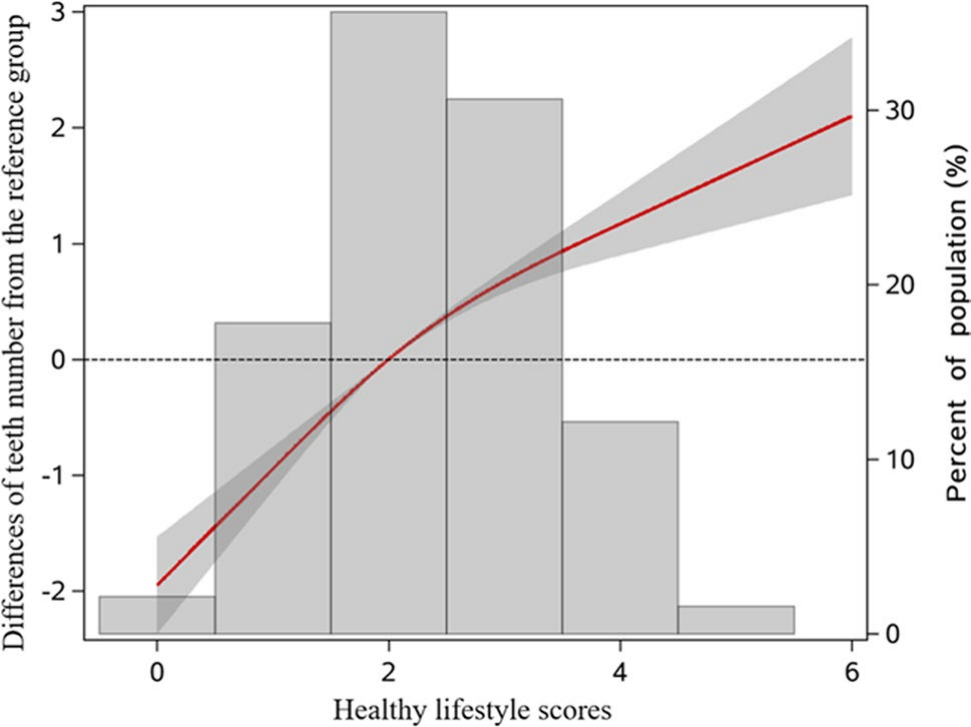
Associations of healthy lifestyle scores with tooth count based on RCS. Note: The lines representing the difference in tooth count were calculated by RCS for the healthy lifestyle scores in the model and the gray color areas represented 95% confidence intervals. The models were adjusted for age, gender, ethnicity, education level, marital status, annual household income, dental visit within the past year, hypertension, diabetes, CKD, CVD, arthritis, respiratory diseases, and cancer

RCS analysis revealed a significant nonlinear association between the healthy lifestyle score and tooth count (*P*_for nonlinearity_ < 0.01). The RCS curve was relatively steep at lower healthy lifestyle scores, indicating a more pronounced increase in tooth count with initial improvements in lifestyle adherence, and then gradually flattened at higher scores (Fig. 2).​

### Stratified analyses​

When participants were stratified into three age groups (20–39 years, 40–64 years, and ≥ 65 years), the association between healthy lifestyle and tooth count varied significantly by age (*P*_for interaction_ < 0.01). No significant association was observed in the 20–39 years group (*P* > 0.05), whereas significant associations were found in the other two groups. Notably, the association was strongest among individuals aged 65 and older (*P*_for interaction_ < 0.01) (Table 3).

**Table 3:** Weighted regression coefficients *β*(95%*CI)* of tooth count associated with healthy lifestyle score across age and gender groups

Subgroup		β(95%CI)	*P*	*P*_for interaction_
Age				< 0.01
18–39 years old				
	Low adherence group	0.00		
	Moderate adherence group	−0.04(−0.31, 0.22)	0.75	
	High adherence group	0.21(−0.07, 0.48)	0.14	
40–64 years old				
	Low adherence group	0.00		
	Moderate adherence group	1.58(0.99, 2.17)	< 0.01	
	High adherence group	2.25(1.52, 2.98)	< 0.01	
65 years old and above				
	Low adherence group	0.00		
	Moderate adherence group	1.80(1.02, 2.59)	< 0.01	
	High adherence group	3.74(2.59, 4.89)	< 0.01	
Gender				0.16
Male				
	Low adherence group	0.00		
	Moderate adherence group	1.37(0.83, 1.90)	< 0.01	
	High adherence group	1.94(1.31, 2.57)	< 0.01	
Female				
	Low adherence group	0.00		
	Moderate adherence group	0.83(0.36, 1.29)	< 0.01	
	High adherence group	1.30(0.75, 1.85)	< 0.01	

The models were adjusted for age, gender, ethnicity, education level, marital status, annual household income, dental visit within the past year, hypertension, diabetes, CKD, CVD, arthritis, respiratory diseases, and cancer

In gender-stratified analyses, healthy lifestyle was positively associated with tooth count in both males and females (all *P* < 0.05). Although the association appeared stronger in males, the interaction between gender and healthy lifestyle was not statistically significant (*P*_for interaction_ = 0.16) (Table 3).

### Weighted associations of different lifestyle scores consisting of four lifestyle factors with tooth count

Upon removing each lifestyle factor from the healthy lifestyle score, the strength of the association was slightly increased when BMI was excluded, but decreased to varying degrees when other factors were omitted. The greatest reduction in the association strength was observed after removing the smoking factor. Specifically, after this exclusion, the *β* coefficients (95%*CI*) were 0.35 (0.08, 0.62) for the moderate-adherence group and 0.23 (-0.06, 0.52) for the high-adherence group (Table 4).

**Table 4:** Weighted associations of different lifestyle scores consisting of four lifestyle factors with tooth count

	β (95%CI)	*P*
Healthy Lifestyle Index (excluding smoking)		
Low adherence group	0.00	
Moderate adherence group	0.35(0.08, 0.62)	0.01
High adherence group	0.23(-0.06, 0.52)	0.12
Healthy Lifestyle Index (excluding alcohol consumption)		
Low adherence group	0.00	
Moderate adherence group	0.86(0.62, 1.11)	< 0.01
High adherence group	1.20(0.90, 1.50)	< 0.01
Healthy Lifestyle Index (excluding diet)		
Low adherence group	0.00	
Moderate adherence group	0.63(0.35, 0.92)	< 0.01
High adherence group	0.83(0.53, 1.12)	< 0.01
Healthy Lifestyle Index (excluding BMI)		
Low adherence group	0.00	
Moderate adherence group	1.22(0.91, 1.54)	< 0.01
High adherence group	1.94(1.60, 2.09)	< 0.01
Healthy Lifestyle Index (excluding physical activity)		
Low adherence group	0.00	
Moderate adherence group	0.84(0.57, 1.11)	< 0.01
High adherence group	1.48(1.13, 1.84)	< 0.01

The models were adjusted for the excluded lifestyle factor, age, gender, ethnicity, education level, marital status, annual household income, dental visit within the past year, hypertension, diabetes, CKD, CVD, arthritis, respiratory diseases, and cancer

## Discussion

This study demonstrated a significant positive association between greater adherence to a healthy lifestyle and a greater number of teeth among the American population. While the direction of this association was consistent across age and gender subgroups, the strength of association was more pronounced in older adults. Notably, the association strength decreased significantly when smoking was excluded from the healthy lifestyle score.

Our findings are consistent with prior research. Previous studies found that maintaining a greater number of natural teeth plays a crucial role in preserving nutritional status, physical function, and cognitive health in older adults, thereby contributing to healthy aging [21, 22]. Based on data from the 4th National Oral Health Survey, another study reported that individuals with healthy behaviors were more likely to retain 20 natural teeth [23]. Iwasaki et al. also confirmed that maintaining multiple healthy lifestyles (non-smoking, physical activity, healthy weight, and high diet quality) exerted a protective effect against tooth loss and periodontitis in older Japanese adults [24]. Collectively, these studies validate the beneficial role of healthy lifestyles in preserving oral health. The results of the RCS analysis further indicated that the RCS curve was steeper at lower healthy lifestyle scores, suggesting that individuals with low healthy lifestyle scores could achieve substantial improvements in oral health status by adopting just 1–2 healthy behaviors. This population has the highest risk of lifestyle-related oral diseases, such as periodontitis and dental caries, and thus even a modest improvement in lifestyle adherence confers clinically meaningful benefits.

The marked reduction in strength of association following the exclusion of smoking from the healthy lifestyle score indicates that smoking is a key contributor to tooth loss. The stronger association observed in males may largely be attributed to the higher smoking prevalence among men. Consistent with this, studies based on NHANES have identified smoking as a major lifestyle factor increasing the risk of periodontitis [9]. Data from the 4th National Oral Health Survey indicate that people who never smoked have a 43% higher probability of retaining 20 natural teeth compared to current smokers [23]. Dietrich et al. also reported that current cigarette/cigar smokers had a 20% higher risk of tooth loss compared to never smokers and former smokers [25]. While the adverse effects of tobacco on respiratory and cardiovascular systems have been extensively documented, its direct impact on oral health should not be underestimated [26–30]. Using human gingival biopsy samples, César-Neto et al. demonstrated that heavy smoking modulates the levels of key biomarkers involved in innate and adaptive immune responses in periodontitis; the underlying mechanism may involve inhibiting critical mediators required for pathogen clearance while exacerbating mediators associated with tissue destruction, ultimately increasing susceptibility to periodontitis [31]. Beyond increasing the risk of oral diseases, smoking also impairs the efficacy of oral disease treatment. For example, in individuals undergoing periodontal maintenance therapy, the recurrence rates of periodontitis were 44.2% in non-smokers, 68.2% in former smokers, and 80.0% in current smokers, with the risk of recurrence decreasing significantly as smoking cessation duration increased [32].

Given that smoking has been identified as a significant contributing factor to tooth loss in our study, and a healthy lifestyle is closely associated with better tooth preservation, these results indicated that smokers may derive greater benefits from adopting a healthy lifestyle compared to non-smokers. Therefore, public health interventions for oral health should prioritize smoking cessation. Despite a continued decline in China’s overall smoking rate, dropping from 28.1% in 2010 to 23.2% in 2024 among the population aged 15 and above, the substantial population base means the disease burden attributable to smoking remains high [33]. To achieve the tobacco control target outlined in the “Healthy China 2030” Blueprint, which aims to reduce the smoking rate in this demographic to below 20%, enhanced public awareness campaigns, an improved smoking cessation service system, and comprehensive health support for smokers are imperative [34]. However, for smokers, following a healthy dietary pattern, limiting alcohol consumption, and maintaining other healthy behaviors can also provide some benefits for oral health. Thus, it is necessary to emphasize the promotion of a comprehensive healthy lifestyle for current smokers. Even for those who cannot immediately quit smoking, improving other lifestyle aspects can also serve as an effective transitional strategy to protect the remaining natural teeth and reduce the risk of further tooth loss.

In addition to smoking, our study identified diet quality and alcohol consumption as factors with relatively large weights in influencing the number of remaining natural teeth. As this was a cross-sectional study, causal inferences regarding the association of healthy lifestyles with tooth count cannot be established. Previous studies have reported that greater tooth loss is significantly associated with a lower HEI [35]. Future research with higher-level evidence, such as cohort studies, is urgently needed to validate our findings. Notably, the HEI is a well-validated measure of overall diet quality. A higher HEI score reflects greater adherence to dietary guidelines, corresponding to a more balanced and adequate nutrient intake, especially sufficient consumption of vegetables, fruits, and protein-rich foods [36]. Increased intake of green leafy vegetables and fruits has been linked to a lower risk of periodontitis and coronal caries [11, 37]. Because tooth loss is primarily caused by periodontitis and dental caries, the protective association of a higher HEI with these two conditions may indirectly contribute to the retention of natural teeth. Furthermore, multiple studies have demonstrated that excessive alcohol intake is significantly associated with tooth loss and clinical attachment loss, which is consistent with and further supports the findings of the present study [38–40].

Stratified analysis revealed a stronger association between healthy lifestyles and tooth count in older adults. The World Health Organization (WHO) has established a dental health standard: adults aged 80 years should retain at least 20 functional teeth. While tooth wear is inevitable with age, tooth loss is primarily driven by pathological factors such as dental caries and periodontitis rather than aging itself. Given the well-established oral-systemic health connection, promoting healthy lifestyles represents a convenient and cost-effective strategy compared to therapeutic interventions. This approach aligns with primary prevention principles, effectively reducing the burden of oral diseases and potentially mitigating multiple other chronic non-communicable diseases, thereby holding significant public health importance.

No significant association was observed in younger adults in the present study, which may be attributable to the distinct etiologies of tooth loss between younger and older populations. Specifically, tooth loss in younger adults is predominantly driven by non-pathological factors such as orthodontic extractions and trauma, whereas the modifiable lifestyle factors examined in this study are important contributors to oral diseases such as periodontitis and dental caries, which were major causes of tooth loss in older adults. However, this does not imply that younger adults cannot benefit from adhering to a healthy lifestyle. Unhealthy lifestyle behaviors, even in early adulthood, exert cumulative adverse effects on dental and periodontal tissues over time, which may manifest as increased risk of oral diseases and subsequent tooth loss in later life.

The strengths of this study include the use of large-scale, nationally representative data from the U.S. NHANES, comprehensive adjustment for sociodemographic and health-related covariates, and rigorous accounting for the complex survey design, which enhanced statistical power and supported the robustness of our findings. However, several limitations should be noted when interpreting the findings. First, as a cross-sectional study based on NHANES, the results only indicate an association between healthy lifestyles and tooth count, with weak causal inference; future validation using higher methodological quality studies is needed. Second, conclusions derived from the NHANES dataset are only applicable to the U.S. population and cannot be generalized to other populations; further validation based on studies of other ethnic groups is required. Third, although the assessment of tooth count, the outcome variable in this study, was conducted using a standardized oral examination protocol performed by clinical professionals, it was not possible to distinguish the underlying causes of tooth loss. Finally, some lifestyle and demographic information included in this study was collected via self-reported questionnaires. Although the reliability of the questionnaires was verified in advance, recall bias remains unavoidable.

## Conclusion

This study observes a significant positive association between healthy lifestyles and tooth count among the American population, with a stronger association observed in older adults. This finding suggests that oral health can be improved through lifestyle management, with older adults deriving greater benefits. Furthermore, smoking has been found to be a significant contributing factor to tooth loss. When formulating future oral health policies and oral disease prevention strategies, priority should be given to promoting smoking cessation, while also considering other healthy lifestyle approaches. 

## Supplementary Information

Supplementary Material 1.

## Data Availability

The datasets generated and/or analysed during the current study are available in the NHANES repository, https://wwwn.cdc.gov/nchs/nhanes/Default.aspx.

## Abbreviations

GBD: Global Burden of Disease
HEI: Healthy Eating Index
NHANES: National Health and Nutrition Examination Survey
NCHS: National Center for Health Statistics
BMI: Body mass index
CVD: Cardiovascular disease
CKD: Chronic kidney disease
SD: Standard deviation
CI: Confidence intervals
RCS: Restricted cubic spline
WHO: The World Health Organization
