# Supplementary material for: Association of healthy lifestyle with tooth count among adults aged 20 years and above: a cross-sectional study
Source: BMC Oral Health. 2026 Apr 13;26:1006. doi: 10.1186/s12903-026-08298-3 (PMC13251100; doi:10.1186/s12903-026-08298-3)
Supplement: Supplementary file 1 — Supplementary Material 1. [file 12903_2026_8298_MOESM1_ESM.docx]

Table S1. Weighted frequencies of participants grouped by healthy lifestyle score

| Characteristics | Total | Healthy lifestyle scores | | |
| --- | --- | --- | --- | --- |
|  |  | 0-1 | 2-3 | 4-5 |
| Gender, No. |  |  |  |  |
| Male | 449601698 | 90610778 | 299284003 | 59706917 |
| Female | 439196155 | 78322279 | 285741548 | 75132327 |
| Ethnicity, No. |  |  |  |  |
| Mexican American | 64704468 | 8880462 | 45130470 | 10693535 |
| Other Hispanic | 43550093 | 7048046 | 30794837 | 5707210 |
| Non-Hispanic White | 638753196 | 96403413 | 415091681 | 127258101 |
| Non-Hispanic Black | 87231691 | 10497152 | 59689030 | 17045508 |
| Other Race | 54558406 | 12010171 | 34319533 | 8228702 |
| Education, No. |  |  |  |  |
| Less than high school | 101078799 | 8081455 | 64132874 | 28864470 |
| High school graduate or equivalent | 196110306 | 18673164 | 129704548 | 47732593 |
| More than high school | 591608748 | 108084625 | 391188129 | 92335994 |
| Annual household income,$, No. |  |  |  |  |
| 0-19999 | 91589621 | 9090243 | 57420244 | 25079134 |
| 20000-44999 | 219258817 | 26185399 | 144302663 | 48770755 |
| 45000-74999 | 220852175 | 30372543 | 147296213 | 43183419 |
| 75000 and above | 357097240 | 69191060 | 236006432 | 51899748 |
| Marital status, No. |  |  |  |  |
| Married | 507587063 | 80031720 | 338971708 | 88583635 |
| Widowed/Divorced/Separated/Never married | 381210790 | 54807525 | 246053844 | 80349422 |
| Hypertension, No. |  |  |  |  |
| Yes | 269667355 | 23327336 | 179622124 | 66717895 |
| No | 619130498 | 111511909 | 405403427 | 102215162 |
| Diabetes, No. |  |  |  |  |
| Yes | 72120945 | 4980199 | 47800001 | 19340745 |
| No | 816676908 | 129859046 | 537225550 | 149592312 |
| Dental visit within the past year, No. |  |  |  |  |
| Yes | 351676771 | 1003(6.14) | 4375(26.36) | 1136(7.08) |
| No | 537121082 | 62889261 | 234245700 | 54541811 |
| CKD, No. |  |  |  |  |
| Yes | 54900196 | 5513969 | 33864820 | 15521407 |
| No | 833897657 | 129325276 | 551160731 | 153411650 |
| CVD, No. |  |  |  |  |
| Yes | 62964059 | 4992999 | 39164786 | 18806274 |
| No | 825833794 | 129846246 | 545860765 | 150126783 |
| Arthritis, No. |  |  |  |  |
| Yes | 223217865 | 23836777 | 144166900 | 55214188 |
| No | 665579988 | 111002468 | 440858651 | 113718869 |
| Respiratory diseases, No. |  |  |  |  |
| Yes | 63423158 | 3747219 | 37584751 | 22091188 |
| No | 825374696 | 131092026 | 547440800 | 146841869 |
| Cancer, No. |  |  |  |  |
| Yes | 91522355 | 12133826 | 57763937 | 21624593 |
| No | 797275498 | 122705419 | 527261615 | 147308465 |
